# Borrowing Nuclear DNA Helicases to Protect Mitochondrial DNA

**DOI:** 10.3390/ijms160510870

**Published:** 2015-05-13

**Authors:** Lin Ding, Yilun Liu

**Affiliations:** Department of Radiation Biology, Beckman Research Institute, City of Hope, Duarte, CA 91010-3000, USA; E-Mail: lding@coh.org

**Keywords:** mitochondrial DNA, DNA replication, DNA repair, RECQ4, Twinkle, PIF1, DNA2, SUV3

## Abstract

In normal cells, mitochondria are the primary organelles that generate energy, which is critical for cellular metabolism. Mitochondrial dysfunction, caused by mitochondrial DNA (mtDNA) mutations or an abnormal mtDNA copy number, is linked to a range of human diseases, including Alzheimer’s disease, premature aging‎ and cancer. mtDNA resides in the mitochondrial lumen, and its duplication requires the mtDNA replicative helicase, Twinkle. In addition to Twinkle, many DNA helicases, which are encoded by the nuclear genome and are crucial for nuclear genome integrity, are transported into the mitochondrion to also function in mtDNA replication and repair. To date, these helicases include RecQ-like helicase 4 (RECQ4), petite integration frequency 1 (PIF1), DNA replication helicase/nuclease 2 (DNA2) and suppressor of var1 3-like protein 1 (SUV3). Although the nuclear functions of some of these DNA helicases have been extensively studied, the regulation of their mitochondrial transport and the mechanisms by which they contribute to mtDNA synthesis and maintenance remain largely unknown. In this review, we attempt to summarize recent research progress on the role of mammalian DNA helicases in mitochondrial genome maintenance and the effects on mitochondria-associated diseases.

## 1. Introduction

**Figure 1:**
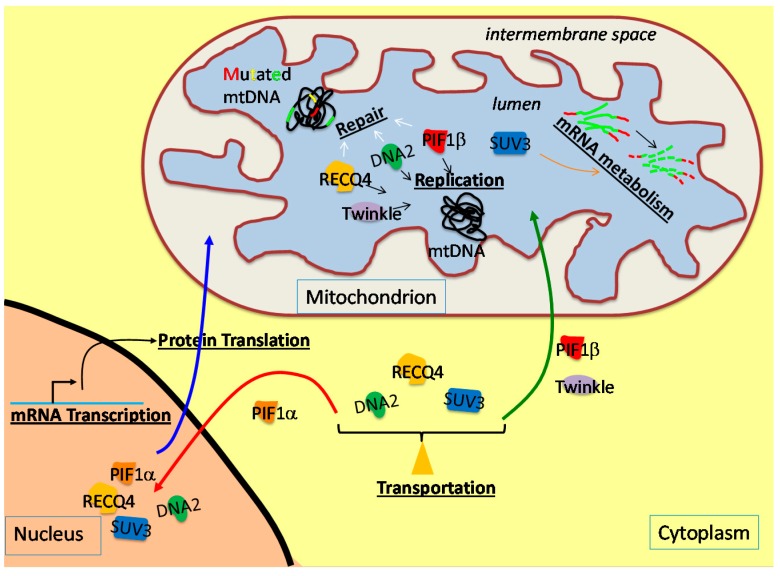
Schematic diagram of the production and the cellular localization of the DNA helicases (Twinkle, purple; RecQ-like helicase 4 (RECQ4), yellow; DNA replication helicase/nuclease 2 (DNA2), green; petite integration frequency 1 (PIF1), red; suppressor of var1 3-like protein 1 (SUV3), blue) that function in the mitochondrion. These DNA helicases are encoded in the nuclear genome, produced in the cytoplasm and transported into the mitochondrial lumen. With the exception of Twinkle, other DNA helicases, including RECQ4, DNA2, PIF1 and SUV3, are transported into the mitochondrial lumen or nucleus depending on the molecular cue. In the mitochondrion, these helicases participate in DNA replication and repair, as well as mRNA metabolism, in order to maintain mtDNA stability.

The mitochondrion, once an autonomous free-living Proteobacterium, became a part of the eukaryotic cell through endosymbiosis approximately two billion years ago [1]. A symbiotic relationship was established, and now, mitochondria not only serve as the powerhouses of the cell by generating adenosine triphosphate (ATP) via oxidative phosphorylation, but also regulate cellular metabolism through synthesizing heme and steroids, supplying reactive oxygen species (ROS), establishing the membrane potential and controlling calcium and apoptotic signaling [2]. Human mitochondria are maternally inherited organelles, which reside in the cytoplasm. The mitochondrial architecture consists of an outer membrane, an inner membrane, an intermembrane space and the matrix or lumen (Figure 1). The mitochondrial number per cell differs from one cell type to another, and each mitochondrion contains multiple copies of the mitochondrial DNA (mtDNA), ranging from one to 15 copies per mitochondrion [3,4]. mtDNA copy number per cell also varies among different tissues due to the tissue-specific epigenetic regulation of the expression of mtDNA replication polymerase γ (Pol γ) [5]. The human mtDNA resides in the lumen and attaches to the inner membrane [6]. The mtDNA forms a small circle, which consists of 16,569 base pairs that encode two rRNA genes, 22 tRNA genes and 13 protein-encoding genes that produce parts of the electron transport chain and ATP Synthase complexes.

mtDNA is thought to be duplicated through either strand-displacement replication or RNA incorporation throughout the lagging strand [7]. Interestingly, mtDNA sequences are highly polymorphic, even within an individual. This is due to the fact that somatic mutations in mtDNA, as a result of replication errors, ROS exposure and aging, make mtDNA sequences different from each other, even within the same cell (heteroplasmic), rather than genetically identical (homoplasmic). To safeguard a healthy population of mitochondria in a cell, mitochondria are constantly dividing (fission) and rejoining (fusion). However, should a pathogenic somatic mutation be introduced into the mtDNA genome, the entire mitochondrial population could be affected. Therefore, to maintain mtDNA stability, it is crucial to ensure faithful mtDNA synthesis. In addition, mitochondria employ several DNA repair pathways to restore DNA integrity in response to damage or replication errors [8]. Failure to do so causes mitochondrial morphological changes [9], which may lead to mitochondrial dysfunction, a phenomenon that has been linked to Alzheimer’s disease and premature aging [10]. Moreover, recent studies have shown that changes in mtDNA copy number are often associated with human cancers [11,12].

DNA synthesis and DNA repair are sophisticated processes that involve multi-protein complexes. Due to the small size of the mtDNA genome and the limited number of genes it encodes, mitochondria have adapted a mechanism to “borrow” enzymes encoded in the nuclear genome for many of its functions, including mtDNA synthesis and repair. For example, DNA helicases are ATPases that break the hydrogen bonds between DNA base pairs and transiently convert double-stranded DNA (dsDNA) into single-stranded DNA (ssDNA), the latter of which can serve as the template for DNA synthesis or allow the repair of damaged bases or nucleotides. These DNA helicases are transcribed in the nucleus, synthesized in the cytoplasm and imported into the mitochondrial compartment. Mitochondrial transport occurs primarily through either the presequence pathway or the carrier pathway. Both pathways involve interactions with the translocase of the outer membrane (TOM) and the translocase of the inner membrane (TIM) protein complexes, though the protein subunits are different for each pathway [13,14,15]. The presequence pathway targets the precursor protein to the lumen, where a mitochondrial targeting signal (MTS) located at the *N*-terminus of the precursor protein is then cleaved by a mitochondrial processing peptidase. The precursor proteins that also express hydrophobic sorting signals are either inserted into the inner membrane or released into the inter membrane space. The carrier pathway usually targets the mitochondrial proteins to the inner membrane, and these precursors have a non-cleavable internal targeting signal (ITS) and form complexes with cytosolic chaperones to prevent aggregation. Nonetheless, there are exceptions to these rules; some proteins, such as the tumor suppressor p53, can also be targeted to the mitochondrion via protein-protein interactions [16].

In mammalian cells, mtDNA replication is promoted by the replicative DNA helicase Twinkle, which is encoded by the *C10orf2* gene in the nucleus. In addition to Twinkle, there are many DNA helicases that contribute to mammalian mtDNA integrity. Interestingly, unlike Twinkle, which is known to exclusively function in the mitochondrion, many of these DNA helicases not only are expressed from the nuclear genome, but also are involved in nuclear DNA replication and repair (Figure 1). This raises several questions. How do these DNA helicases balance their distribution and function in the nucleus and mitochondrion? What triggers the translocation of these helicases between different cellular compartments? How do Twinkle and other helicases collaborate in mtDNA replication and repair? In this review, we summarize recent findings on how these nuclear-encoding DNA helicases contribute to mtDNA integrity and associated diseases, and we will try to shine light on future studies in this active field.

## 2. Mitochondrial Replicative Helicase, Twinkle

**Figure 2:**
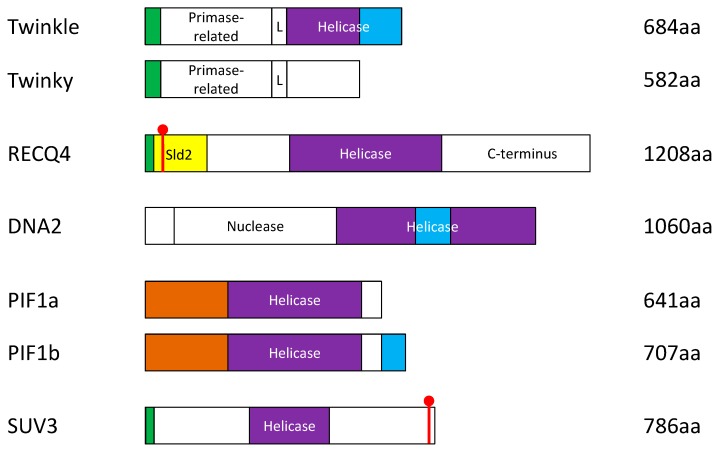
Schematic diagram of the protein domains and alternatively-spliced variants of the human DNA helicases that have known functions in the mitochondrion. Green: mitochondrial targeting sequence (MTS). Red: nuclear localization signal (NLS). Purple: helicase domain. Blue: non-MTS sequence required for mitochondrial localization. Brown: arginine-rich region where potential NLSs reside. Yellow: unique sld2-like domain. L = linker region.

Twinkle (for the T7 gp4-like protein with intramitochondrial nucleoid localization or PEO1) was first identified based on a sequence homology search as T7 gene 4 primase/helicase in 2001 [17]. Although Twinkle is conserved in many eukaryotes, such as the mouse, *Drosophila* and zebra fish, it has no orthologs in yeast [18]. It is possible that other yeast helicases compensate for its role in mtDNA replication. Twinkle is essential for embryonic development in mammalian systems, and it is known to unwind mtDNA for mtDNA synthesis by Pol γ [19]. Immunofluorescence microscopy has revealed that Twinkle proteins form punctate foci within mitochondria and colocalize with mitochondrial nucleoids [17], which are aggregates containing mtDNA and proteins that enact mitochondrial genome maintenance and transcription [20,21]. These foci resemble twinkling stars [17]. Human Twinkle, a 684 amino-acid (aa)-long polypeptide with a molecular weight of 77 kDa, oligomerizes to form a hexamer and exhibits 5'–3' helicase activity due to the conserved superfamily 4 (SF4) helicase domain located at its *C*-terminus (Figure 2) [22]. In addition to the conserved SF4 domain, Twinkle also contains a 42-aa MTS for mitochondrial targeting and a non-functional *N*-terminal primase-like domain that connects to the SF4 domain by a linker domain (Figure 2) [22,23]. The linker region is important for the hexamerization of Twinkle and its DNA helicase activity [24]. Recent studies have also found that Twinkle exhibits DNA annealing activity, indicating a possible involvement of Twinkle in recombination-mediated replication initiation or the fork regression pathway of DNA repair [25]. Interestingly, an alternatively-spliced product, Twinky, lacks part of the *C*-terminus, exists as monomers and has no enzymatic activity [23]. The function of Twinky remains unclear, as it cannot localize to the mitochondrial nucleoids [17] nor associate with Twinkle [23], despite the fact that Twinky contains the proposed MTS at the *N*-terminus (Figure 2). This suggests that the unique *C*-terminus of Twinkle may contain an additional sequence that is also important for its mitochondrial localization. Recombinant human Twinkle, combined with Pol γ purified from insect cells, is sufficient to form the minimal mammalian mtDNA replisome [26]. The hexameric Twinkle ring can efficiently bind to the single-stranded region of a closed circular DNA without a helicase loader and support DNA synthesis by Pol γ through the duplex region [26]. The helicase activity of Twinkle is stimulated by mitochondrial single-stranded DNA-binding protein (mtSSB) [22,27].

Given the essential role of Twinkle in mtDNA synthesis, mtDNA stability is greatly influenced by the Twinkle expression level in cells. For example, overexpression of the wild-type Twinkle is associated with increased mtDNA copy number in skeletal muscle in mice and reduced ROS-induced mtDNA mutations [28,29], whereas depletion of the Twinkle protein by small interfering RNA (siRNA) leads to a significant decrease in mtDNA copy number [30]. Furthermore, increasing evidence has linked a set of mutations, which change the stability and enzymatic activity of Twinkle [31], to a wide range of diseases [32], such as mitochondrial myopathy [33] and autosomal dominant progressive external ophthalmoplegia (adPEO) [34]. Individuals suffering from adPEO bear multiple deletions in their mitochondrial genome and exhibit multiple symptoms, including muscle weakening, hearing loss, nerve damage and Parkinsonism [35,36].

## 3. The Involvement of the Nuclear DNA Helicases

### 3.1. RecQ-Like Helicase 4 (RECQ4)

The gene that encodes RecQ-like helicase 4 (RECQ4) was first identified and cloned based on its limited sequence homology to the highly-conserved RECQ family of superfamily 2 (SF2) DNA helicases [37]. RECQ4 mutations were later identified in patients suffering from Rothmund-Thomson syndrome (RTS), Baller-Gerold syndrome and RAPADILINO (RAdial hypo-/aplasia, PAtellae hypo-/aplasia and cleft or highly arched PAlate, DIarrhea and DIslocated joints, LIttle size and LImb malformation, NOse slender and NOrmal intelligence) syndrome, with phenotypes ranging from premature aging to cancer predisposition [38]. *In vitro*, purified recombinant RECQ4 proteins exist as multimeric proteins and unwind DNA in a 3'–5' direction [39]. Interestingly, RECQ4 not only unwinds DNA, but also exhibits strong DNA annealing activity [40]. In addition to the conserved SF2 helicase domain, the vertebrate RECQ4 contains a unique Sld2-like *N*-terminus (Figure 2) that resembles the essential yeast DNA replication initiation factor Sld2 [41]. Researchers have shown that RECQ4 forms a chromatin-specific complex via this Sld2-like *N*-terminal domain with the MCM2-7 replicative helicase complex and participates in nuclear DNA replication initiation [42,43,44,45,46,47]. This function explains why *recq4* knockout results in embryonic lethality in mice. Furthermore, the expression of a RECQ4 fragment containing only the Sld2-like *N*-terminal domain is sufficient to support embryonic development [48,49]. The *C*-terminus of RECQ4, which is highly conserved among vertebrates, contains a putative RecQ-*C*-terminal domain (RQC) [50]. Although this *C*-terminal domain is not required for unperturbed DNA replication, a recent study suggests that it is crucial for replication elongation when cells are exposed to ionizing radiation [51]. It has been demonstrated with other members of the RECQ family helicases that the RQC domain is important for the DNA unwinding activity [52]. Therefore, it is possible that the helicase activity of RECQ4 is involved in stabilizing or repairing the damaged replication forks. Because many disease-associated RECQ4 mutations disrupt the conserved *C*-terminal domain [38], understanding the potential function of RECQ4 in replication fork stability in response to ionizing radiation may provide important insight into the pathogenicity of these diseases.

In addition to affecting nuclear DNA replication, RECQ4 expression level also affects mtDNA copy number [53]. Consistent with this, RECQ4 also localizes to the mitochondrion [16,53,54,55,56], and the existence of a MTS within the first 20 aa has been proposed [16]. That said, whether RECQ4 is targeted to the mitochondrion via the conventional MTS remains to be validated. Given that RECQ4 interacts with the nuclear DNA replicative helicase complex and plays a critical role in nuclear DNA replication [42,43,44,45,46,47], it is possible that RECQ4 might have a similar role in mtDNA synthesis. Indeed, in a recent study from our laboratory, we reported a weak interaction between RECQ4 and the mitochondrial replicative helicase Twinkle that can be detected in human whole-cell extracts [56]. Perhaps most surprisingly, we found that this interaction between RECQ4 and Twinkle was significantly enhanced in human cells carrying the most common lymphoma-prone RECQ4 mutation: c.1390+2 delT. This mutation produces RECQ4 polypeptides lacking Ala420-Ala463 residues immediately upstream of the conserved helicase domain [56]. As a consequence, there is increased mtDNA synthesis, leading to an increase in the mtDNA copy number and mitochondrial dysfunction in these cells. Clearly, residues Ala420–Ala463, which are missing in this cancer-prone RECQ4 mutant, have an important inhibitory role in mtDNA synthesis, and we further elucidated how this regulation works. We found that residues Ala420–Ala463 of RECQ4 are required for the interaction with p32, and this interaction negatively regulates RECQ4 mitochondrial localization [56]. p32, which resides in both the mitochondrion and the nucleus [56,57], is involved in regulating mitochondrial innate immunity [58], energy production [57,59] and mitochondrial protein translocation [60]. Cells expressing RECQ4 mutants that lack these 44 aa show defective RECQ4-p32 interactions, increased RECQ4 mutant proteins in the mitochondrion and decreased nuclear RECQ4, suggesting that the excess of RECQ4 molecules in the mitochondrion likely results from increased nuclear-mitochondrial transport [56]. Therefore, this work presents a model for the mechanism used by cells to balance the distribution of RECQ4 in the nucleus and mitochondrion via direct protein-protein interaction.

Although mitochondrial localization of RECQ4 is restricted by p32, RECQ4 itself has also been suggested to function as a positive regulator of the mitochondrial transport of the p53 tumor suppressor via a direct protein–protein interaction [16]. Interestingly, mitochondrial localization of p53 can be blocked by the chaperone protein nucleophosmin (NPM) [61], which was found to also interact with RECQ4 in the nucleoplasm [56]. Although the domain of RECQ4 that interacts with NPM remains to be determined, it is tempting to speculate that NPM inhibits the mitochondrial transport of p53 via its interaction with RECQ4 in the nucleus. In summary, RECQ4 is a dynamic interacting protein, and its protein-protein interactions not only govern the rate of nuclear and mitochondrial DNA synthesis, but also regulate its cellular localization.

### 3.2. DNA Replication Helicase/Nuclease 2 (DNA2)

The DNA replication helicase/nuclease 2 (*DNA2*) gene was first isolated from a genetic screen in budding yeast [62], and the human *DNA2* gene was later identified based on its sequence homology to the yeast counterpart [63]. Human DNA2, a 120-kDa polypeptide, has two independent functional domains: the *N*-terminal nuclease and the *C*-terminal helicase domain (Figure 2). DNA2 is highly conserved among the eukaryotes. As such, expression of either the human or *Xenopus laevis* DNA2 complements the temperature sensitivity of a *DNA2* (*DNA2-1*) mutation in budding yeast [64]. However, the specificity of DNA2 enzymatic activity may vary across species due to the widely divergent sequences of the distal *N*-terminal regions [65,66]. DNA2 proteins purified from human cells and insect cells show that the nuclease domain has both 5'–3' and 3'–5' nuclease activities [65,67], whereas the helicase unwinds dsDNA that contains a 5' flap as a tail [67]. Data from yeast studies suggest that the 5'–3' helicase activity of DNA2 facilitates the production of a 5' flap structure, a substrate of DNA2 nuclease activity *in vitro* [68]. Nonetheless, the yeast helicase activity *in vivo* is dispensable for cell growth under normal conditions [69,70]. It remains to be determined if this is also the case in higher eukaryotes and if other DNA helicases can compensate for the DNA2 helicase activity.

In the nucleus, DNA2 interacts with proliferating cell nuclear antigen, also known as PCNA, a protein that is important for replication processivity and prevents the accumulation of DNA double-strand breaks (DSBs) during replication [71]. In addition, DNA2 interacts with the Fanconi anemia complementation group D2 (FANCD2) protein and functions in the FANCD2-dependent interstrand crosslink repair pathway [72]. Cells with depleted DNA2 show increased DSBs [71], internuclear chromatin bridges [73] and increased sensitivity to interstrand crosslinking agents due to a reduced homologous recombination frequency [72]. Furthermore, DNA2 participates in long-range DNA resection, in concert with the Werner syndrome ATP-dependent helicase (WRN) and the Bloom syndrome protein (BLM), in DSB repair [74,75,76]. DNA2 also stimulates BLM helicase activity [75]. Recently, DNA2 was also implicated in telomere maintenance based on its ability to cleave G-quadruplex DNA, and heterozygous DNA2 knockout mice were found to be prone to telomeric DNA damage and aneuploidy [77].

Although DNA2 can localize to the nucleus and play a role in nuclear DNA repair, immunofluorescence microscopy data suggest that the majority of the DNA2 molecules are found in the mitochondrion [73,78]. DNA2 does not contain a classical MTS/ITS, but its localization to the mitochondrion requires the sequence located within 734 and 829 aa [78]. It remains unclear how cells regulate the distribution of DNA2 in the mitochondrion and the nucleus in response to either the cell cycle or DNA damage. DNA2 interacts with and stimulates Pol γ in the mitochondrion and is thought to also function in concert with flap structure-specific endonuclease 1 (FEN1) to process 5'-flap intermediates and participate in repairing oxidative lesions in mtDNA by long-range base excision repair [78]. Indeed, DNA2 proteins colocalize with mtDNA nucleoids and Twinkle and through an unknown mechanism; this localization increases in cells carrying some of the adPEO-associated Twinkle mutations [73]. Interestingly, point mutations in the *DNA2* gene itself have also been linked to adPEO, and these patients show progressive myopathy with mitochondrial dysfunction [79]. Importantly, one mutation located within the helicase domain altered the DNA unwinding efficiency [79], suggesting that the helicase activity of DNA2 has an important role in mtDNA maintenance in humans. Therefore, similar to Twinkle, DNA2 is important for maintaining healthy mitochondrial DNA and preventing related diseases.

### 3.3. Petite Integration Frequency 1 (PIF1)

PIF1, which stands for petite integration frequency 1, is conserved in both budding yeast and humans [80,81,82,83]. PIF1 is a member of the superfamily 1 (SF1) helicase family and has 5'–3' DNA unwinding activity (Figure 2) [84,85,86]. Similar to RECQ4 and DNA2, PIF1 localizes to both the nucleus and the mitochondrion [83]. However, unlike RECQ4 and DNA2, PIF1 mitochondrial localization in human cells is regulated by alternative splicing, which produces α and β isoforms [83]. Both the α and β isoforms contain the intact helicase domain and the *N*-terminus (Figure 2), which has arginine-rich nuclear localization signals [83] and is important for the interaction with ssDNA [84].

The PIF1 α isoform consists of 641 aa and has a short distal *C*-terminus. This isoform localizes to the nucleus [83], and PIF1 function in the nucleus has been extensively demonstrated [83,85,87,88]. The expression of PIF1 is cell cycle regulated, and the downregulation of PIF1 leads to cell cycle delay [81,83]. Both yeast and human PIF1 bind DNA and promote DNA replication through interaction with G-quadruplex DNA regions [86,87,89,90,91,92]. This activity is important for maintaining telomere integrity and for resolving stalled replication forks [85]. Reduction of PIF1α by siRNA knockdown decreases cancer cell survival, but has no impact on non-malignant cells [93], and this is likely due to its role in restarting stalled replication forks [85,94].

The PIF1 β isoform (707 aa) has a long distal *C*-terminus with a lipocalin motif (protein secretion signal; Figure 2). This *C*-terminal region results from alternative splicing and is not present in the α isoform. PIF1β is expected to have similar biochemical properties, compared to PIF1α, as they contain the same helicase domain. However, unlike the α isoform, the majority of this β isoform localizes to the mitochondrion, with some residual nuclear signal [83]. Evidence from yeast studies suggests that PIF1 may associate with mtDNA and mitochondrial inner membranes [95] and contribute to reducing DSBs in mtDNA [96]. Furthermore, it is required for repairing UV- and ethidium bromide-damaged mtDNA [80]. In addition, Twinkle, which cannot efficiently unwind G-quadruplex DNA [97], may rely on PIF1 helicase activity to remove G-quadruplexes, which could potentially lead to mtDNA deletions. Nonetheless, it is unknown how the distal *C*-terminus, which is unique to PIF1β, promotes its mitochondrial localization and how PIF1β protects mtDNA from DSBs. Interestingly, deletion of *PIF1* rescued the lethal phenotype of DNA2 in budding yeast, suggesting that PIF1 and DNA2 may be involved in similar, but non-redundant pathways in the mitochondrion [98].

### 3.4. Suppressor of Var1 3-Like Protein 1 (SUV3)

SUV3, a member of the DExH-box helicase family, was first identified in budding yeast as the suppressor of var1 (the small subunit of mitochondrial ribosomal protein) [99], and the gene was later found to be conserved in humans [100]. *SUV3* knockout mice are embryonic lethal, whereas heterozygous mice have shortened lifespan and develop tumors at multiple sites, due to a reduced mtDNA copy number and an elevated number of mtDNA mutations [101]. Reduced SUV3 expression was observed in human breast tumor samples [101]. Nonetheless, unlike RECQ4, PIF1 and DNA2 helicases, the effect of SUV3 deficiency on mtDNA copy number and stability is likely indirect. For example, in the mitochondrion, SUV3 forms a complex with polynucleotide phosphorylase (PNPase) to function in mtRNA degradation [102]. Indeed, analysis using purified recombinant human SUV3 proteins demonstrated that SUV3 is an active ATPase and capable of unwinding not only DNA, but also RNA in a 3'–5' direction [102,103,104]. This SUV3-PNPase complex transiently associates with the mitochondrial polyadenylation polymerase when the inorganic phosphate level is low in the mitochondrial lumen [105]. The three-component complex is capable of regulating the length of the RNA poly(A) tail. Consistent with this, siRNA knockdown leads to an increase in the amount of mtRNA with shorter poly(A) tails, a reduction in mtDNA copy number [106] and an increase in the rate of apoptosis [107]. In addition, expression of a mutant defective in the ATPase function leads to an abnormally high level of mtRNA, due to the slow mRNA turnover rate [108]. Although it remains unclear how a defect in mtRNA degradation contributes to mtDNA instability in SUV3-deficient cells, it is possible that the abnormal level of mtRNA imposes cellular stress, leading to overproduction of ROS and mtDNA damage.

Early studies suggest that SUV3 localizes to the lumen of the mitochondrion, presumably through cleavage of an MTS localized at the distal *N*-terminus (Figure 2) [103,107]. However, recent studies provide evidence that SUV3 also localizes to the nucleus with a potential nuclear localization signal located between residues 777 and 781 at the *C*-terminus [104,107]. In the nucleus, SUV3 interacts with nuclear DNA replication and repair factors, such as the RECQ helicases BLM and WRN [109], as well as replication protein A (RPA) and FEN1 [104]. Therefore, it is possible that, at least in humans, SUV3 is a key player in nuclear genome maintenance due to its participation in DNA damage repair, whereas it maintains mitochondrial genome integrity by participating in mtRNA metabolism. The reason why cells utilize an mtRNA helicase in nuclear DNA damage repair remains unknown. Interestingly, in mammalian cells, there is an increase in the degradation of mtRNA, but not cytoplasmic RNAs, to protect cells in response to oxidative stress [110]. It is possible that the involvement of SUV3 in nuclear DNA repair provides a mechanism for cells to “sense” oxidative DNA damage and induce mtRNA degradation. Therefore, identifying the molecular switch that balances the localization and the two distinct functions of SUV3 might reveal a novel crosstalk between the nucleus and the mitochondrion in response to DNA damage.

## 4. Conclusions

Given that mitochondria provide the vital ATP energy source needed by diverse cellular processes that support the development of an organism, it is not surprising that abnormal mtDNA copy number and mitochondrial dysfunction have been correlated with a decline in tissue maintenance and regeneration. Tissue degeneration may contribute to some of the symptoms, such as muscle weakening, hearing loss, nerve damage and Parkinsonism observed in the adPEO1 patients [35,36]. Growing evidence also suggests a close association between mitochondrial dysfunction and age-related bone diseases. For example, osteoporosis is a result of the loss of bone mass and is one of the common symptoms associated with aging [12,111]. Studies in mice indicate that increased apoptosis in osteoblasts, due to the accumulation of ROS generated by damaged mitochondria, is one of the main causes of bone loss [112]. Interestingly, RTS patients with RECQ4 mutations show abnormal bone development and osteoporosis at an early age [113]. Therefore, it is possible that these RTS-associated RECQ4 mutations lead to mitochondrial dysfunction and contribute to the premature aging phenotypes.

In addition to their association with tissue degeneration and developmental defects, mitochondria have recently gained attention for their potential use both as diagnostic tools and as therapeutic targets for cancer treatment [11]. Variations in mtDNA copy number are observed in many cancers and correlate with tumor aggressiveness and survival outcome. For example, mtDNA copy number is significantly elevated in various types of lymphoma, including Burkitt lymphoma and non-Hodgkin lymphoma [114,115,116,117]. In addition, highly invasive osteosarcoma cells contain enlarged mitochondria and larger amounts of mtDNA, and inhibiting replication of mtDNA in these cells also effectively slows down tumor growth [118,119,120]. Because mtDNA copy number correlates with cell growth [121], deregulated mtDNA synthesis could be a risk factor that contributes to cancer pathogenesis or that sustains cancer cell growth. Therefore, reducing aberrant mtDNA synthesis in cancer by targeting enzymes involved in mtDNA synthesis or mtDNA repair may be an effective strategy for controlling tumor progression. It would be of great interest for future studies to explore the possibility that the DNA helicases we have summarized here may be cancer drug targets or biomarkers for cancer diagnosis and prevention.
